# New Preparations of Gold for Stopping Carious Teeth

**Published:** 1851-10

**Authors:** John Tomes

**Affiliations:** Surgeon Dentist to the Middlesex Hospital.


### SELECTED ARTICLES.

### ARTICLE XII.

*New Preparations of Gold for Stopping Carious Teeth.* By JOHN TOMES, F. R. S., Surgeon Dentist to the Middlesex Hospital.

IF the various operations performed by the dentist were compared, with a view to ascertain the relative value of each, it is probable that the stopping of carious teeth would be placed first on the list. A tooth skilfully plugged under favorable circumstances, is, in point of durability and usefulness, scarcely inferior to one that has not been stopped, or to the tooth had it not been attacked with caries, and needed plugging. Indeed, a tooth which has been well plugged before disease had too far reduced its substance, is in effect restored to the condition of a sound and healthy organ.

If inquiry be made of those who have been accustomed to attend to their teeth, and are somewhat advanced in years, many instances may be found of teeth which have been stopped twenty, thirty, and even forty years; but teeth which began to decay, and yet have remained useful for such lengthened periods without having been plugged, will not be found.

The knowledge of these facts has for many years past led dentists to pay great attention both to the performance of the operation, and to the material most suitable for stopping teeth; and the results of individual experience have been from time to time published.

It may, I think, be safely said that all experienced dentists regard gold as by far the best material for filling teeth, and deprecate the use of any substitute when that metal can be effectively used.

The gold has been used in the form of foil reduced from plate by hammering to suitable degrees of thickness; and in the preparation of this, great care and expense have been bestowed. The superiority of gold over all other substances has for a long time been acknowledged; but there are many difficulties in its use, and these require considerable practice and skill to overcome. The gold may be good, and the case favorable; yet, in the absence of dexterity in the operator, the filling may be, and frequently is, defective. Neither will this appear astonishing when the conditions which constitute a good plug are considered in connection with the position of the teeth, and the parts of these which may require plugging. A perfectly successful plug is a solid mass of gold, fitting perfectly the cavity in the faulty tooth. Hitherto, gold foil has alone been used. This is introduced into the cavity in various forms—in long strips folded up and down from the top to the bottom of the cavity; in small masses, introduced one after the other; or in small carefully-made cylinders. Whichever method is practised, great care is taken to press the gold against the sides of the cavity in the process of filling, as pressure on the surface of the plug condenses the gold only for a very short distance below the surface. If, for instance, the masticating surface of a molar tooth which has a large hole in it be plugged with gold foil, forced in from the surface, and pressed only towards the bottom of the cavity, it will be found, on removing the tooth, that the gold near the surface has been compressed into a hard scale, while that occupying the bottom of the cavity is porous, and admits of further compression. Hence it has been found necessary to pack in gold, and in the steps of the process to compress it against the sides of the cavity. When the cavity has been in this manner filled, wedge-shape instruments are forced into the gold, and as room is thus made, more of the metal is added, until the instrument can no longer be made to enter the plug. Then the surface of the plug is compressed forcibly with the plugging instrument, the superfluous gold is filed away, and the surface of the plug burnished. Now, if the operation has been conducted in the best possible manner, and the gold used unexceptionable, the plug will be a perfectly solid mass; the foil will have become cold welded. This result has been obtained, and is, perhaps, not very unfrequently obtained by most good operators ; but the result would be much more constant if the gold foil having the welding property could at all times be obtained, and the cavities of diseased teeth were favorably situated for applying the requisite amount of compression. We cannot, however, choose the situation of the cavities ; we must treat them in whatever position they occur. But something can be, and, I believe, I may safely say has been done towards the improvement in gold. A gold has been produced, which, under the pressure of the plugging instrument, welds with the greatest readiness into a solid mass capable of being rolled into plates or drawn into wire.

Platinum is obtained in a spongy condition, is heated, and then compressed in a solid mass ; it, in fact, welds as wrought iron does when hammered at a white or welding heat, as it is called. The new preparation of gold may, with propriety, be called spongy gold, from its close resemblance as regards condition, to spongy platinum. But the gold welds under pressure without the aid of heat.

In 1825, Mr. Caudius Ash obtained a precipitate of gold by using copper, which he thought would do for stopping teeth. He tried it, and did not find that it answered his expectation.

The idea was abandoned without further effort. Had he continued his experiments, it is probable he might have obtained a result which would have encouraged him to proceed; and, judging from the success which has attended his untiring efforts in the improvement of mineral teeth, it can scarcely be doubted but that he would have produced a gold similar to that which has recently been presented.

Some months since, a Parisian dentist, on his return through London from a visit to America, brought with him two or three teeth plugged with spongy gold. He called on several dentists—myself amongst the number—and exhibited the stopped teeth. This gentleman also called on Mr. Ash, and offered to sell him the secret of preparing the gold, with directions for its use, both of which he had learned in America. While negotiations were going on, the method of preparation was sold to a dentist for a large sum, and the conditions of sale were considerably raised to Mr. Ash, who then declined the offer.

The purchaser, I am told, found that the gold prepared in accordance with the directions he received did not at all answer his expectations, and he (for the time) abandoned the idea of using it. Mr. Ash, however, recollecting his former experiment, again commenced to work on the subject, and succeeded in producing a preparation of gold which readily welded. Of this he sent me half an ounce. It consisted of a fine brown powder, and of small masses, which readily broke down into powder when pressed lightly. Under the microscope, the powder was seen to consist of minute particles of bright metallic gold, in the form of irregular crystals or nodules. Under pressure, these minute particles readily unite into a solid mass. Several teeth were plugged, by introducing small balls of the powder and compressing it from the exposed surface of the plug. The results seemed highly satisfactory; but on removing the plug it was found that the welding extended but a short distance from the surface against which the instrument was applied, while the lower part of the plug remained porous, and maintained the brown color of the powder. This condition indicated a serious imperfection in the method of using the metal, and other plans were tried: teeth were partially filled with the powder, and this was condensed by forcing it towards the circumference of the cavity, thereby forming a solid ring; more powder was then added, and the plug completed. By these means a perfectly solid plug was formed, to which no objection could be made. In practice, however, the use of the powder was attended with considerable difficulty, even though introduced into the teeth in lumps, in consequence of the great waste. More or less constantly escaped into the mouth before its condensation could be effected. In stopping teeth in the upper jaw the waste was very great. It occurred to me that this difficulty could be overcome by wrapping the gold in foil. This experiment was tried, and the result equalled my expectation. By this method I succeeded in making very perfect plugs, which, when removed, presented a solid uniform mass of metal, perfectly welded, and free from pores.

Still the gold seems to admit of improvement—in fact, requires to be produced in the shape of a sponge instead of a powder, so that the present difficulties of manipulation might be overcome, and the use of gold foil rendered needless.

In the *American Journal of Dental Science* for October 1850, the following editorial notice will be found:—"*A new preparation of gold for filling teeth.*—Within the last few weeks several dentists in New York have tested the value of sponge gold, as a substitute for gold foil, for filling teeth; but to what conclusion they have arrived with regard to it we have not been able to ascertain. We have been informed, however, that some are disposed to think very favorably of it.

"The preparation is of a reddish brown color, and readily crumbles into a fine powder on being rubbed; but, on being pressed together, it at once becomes a compact mass. A few weeks ago we received from the manufacturer, Dr. Maire, through Dr. G. E. Hawes of New York, a small quantity of it, with a request that we would try it. This we have done; but our experiments have been too limited to enable us to say much with regard to its value. We tried it in the cavity of a dead tooth out of the mouth, and succeeded in making a very solid filling. The surface assumed the color of gold, and presented a highly polished and beautiful appearance; but, on removing it from the tooth, we found that every other part retained the reddish brown color of the sponge, with here and there the lustre of a particle of gold. For front teeth this would constitute an insuperable objection. On breaking it, the fractured surfaces had the same appearance. We have also seen two incisors that were filled with it, which were correspondingly discolored.

"It may, perhaps, answer a very good purpose for filling the grinding surface of a molar tooth; but, as it crumbles into powder so readily, we should not think it could be employed for filling a small cavity in the approximal surface of any tooth where the aperture between it and the adjoining organ was very narrow. It is possible, however, that some may have discovered a method of introducing it into the cavity of a tooth of which we are ignorant; and, as our experiments with it have been very limited, we would be glad if some of our brethren of New York, who have given it a thorough trial, would favor us with their opinion with regard to its value."

The gold here described is probably similar to that prepared by Mr. Ash; but, either from the manner of plugging, or from the quality of the gold, the results seem to have been less satisfactory than those I have obtained. The two subsequent numbers of the Journal contain no further information on the subject.

Mr. George, in conversation, told me that he had prepared some spongy gold by making a plate largely alloyed with silver, and then dissolving out the latter metal with nitric acid. In the first experiment he succeeded in producing a gold which formed a good plug; but, in several subsequent trials, he had failed to obtain a similar result.

Some years since, I read an account of precipitating gold from its solution by oxalic acid; and on trying the experiment, found that part of the gold was thrown down in a thin sheet. It did not occur to me at the time to use this for stopping teeth; but when the spongy gold was talked of, the experiment recurred to me, and I mentioned the circumstance to my friend Mr. Makins of Kingston, and also showed him the results obtained by Mr. Ash. Mr. Makins and myself were students together at King's College, and colleagues at Middlesex Hospital, he having a short time since held the chemical chair in the school attached to that institution. I knew that, since his retirement from the lectureship, Mr. Makins had been working in metals. At my request he at once undertook to make some researches on the forms in which gold could be obtained, and the results have been most satisfactory.

Mr. Makins has obtained a soft and malleable gold in two forms—one a thin leaf, another a soft compressible sponge.

The leaf has a frosted appearance, which a microscopic examination shows to arise from the surface being minutely nodulated with here and there irregular, imperfect, crystalline columns, arising to a considerable height from the surface of the leaf. If two of these leaves are pressed together, even with an ivory paper-knife, they cannot be again separated. This gold, introduced into a small cavity, condenses under moderate pressure into a most perfect plug; but, if the cavity be large, it must be introduced in small pieces, and each one compressed before introducing another, much in the same way as in using gold foil, excepting that much less care is required as regards the packing and the direction of the folds of the metal; for, with moderate care, the whole may be welded into a compact uniform mass.

The sponge is revealed by the microscope to be a porous mass of bright metallic gold. It may be compressed by the thumb and finger to a third of its bulk, or may be torn into small pieces without exhibiting any tendency to fall into powder. The smaller pieces may again be united into a mass by simply pressing them together in the hand. These are advantages which no other form of gold possesses.

The sponge, if the bulk be not too great, condenses into a compact solid mass, when pressed into the cavity of a tooth with the ordinary plugging instruments, and presents a bright burnished surface where the steel instrument has come in contact with it, and a hard frosted surface when it lies in contact with the surface of the cavity. If the plug be sawn through, the interior will be found to be compact, and the angles formed by dividing a cylindrical mass will be found to be sharp and hard. Such a plug made with gold foil may present a similar appearance; but, commonly, the angles will be much less firm, and the plug will fall into several pieces, corresponding to the portions of foil which were separately introduced.

In estimating the relative value of the new forms of gold for stopping teeth, as compared with the gold foil in common use, it must be remembered, that the latter has a smooth, more or less burnished surface; that such surfaces weld with difficulty while the former have nodulated surfaces which have not been burnished or subject to pressure, and that they weld readily; that the foil requires very careful packing, both as regards the position of the folds and their size; while the new gold, from the readiness with which it welds, needs no such care.

The properties of the new and old forms of gold may be shown by taking an equal mass of each, and, with a plugging instrument, pressing them upon the surface of a six-pence. Each will assume the form of a plate; but the foil will break up into small fragments and layers when bent and pressed with the finger nail; while the sponge and leaf will present a hard rigid mass, which with force may be broken, but it will not fall into small fragments or break up into layers. Again, if the surfaces of two pieces of the new gold which have been pressed against the coin, be placed in contact, and firmly pressed together, they will adhere—a condition which will not be found to obtain with the pieces of gold foil similarly treated. If the surfaces of the plates of the new gold are highly burnished with the stopping instrument, and are then pressed together, they will adhere, but very imperfectly, if at all.

In giving directions for using the new forms of gold, it will be unnecessary to urge the absolute necessity of removing all the softer dentine from the cavity of the tooth to be plugged, and to again state what should be the form of the cavity for receiving the gold, as these points have been fully dwelt on by many writers, and by myself, when treating on plugging teeth, in my Lectures on Dental Physiology and Surgery; for the form of cavity should be similar, whatever kind of gold be used: it should approach a cylinder as nearly as is practicable; and overhanging edges of enamel should, if possible, be removed.

If the cavity be of moderate size, and shallow, (for instance not more than the sixteenth of an inch in depth,) sufficient sponge gold for completing the plug may be taken, and pressed with a plugging instrument into the cavity; it should then be gradually worked round the circumference of the cavity, with an instrument having a small extremity. At first the pressure should be light, but afterwards it should be increased till the plug is consolidated. At this stage of the operation, the plug (if sufficient of the sponge has been taken) will project above the orifice of the cavity. The surface may now be cut or filed away, and the plugging instrument again used. The gold will probably sink a little under the reapplication of the instrument, in consequence of the removal of the surface, which had become so hard that the pressure had not operated through it. The greater hardness of the surface would seem to indicate that the plug is imperfect, from the unequal density in the different parts. It is not so, however. If a piece of brass (or any other metal, indeed) be hammered, it will, in working, be found to be harder near the hammered surface than in the interior. Yet the interior will be harder than if the brass had not been hammered, and it will be hard enough. So with a gold plug, if the surface is the hardest, yet the interior will be hard enough, and to the eye will be perfectly compact. Indeed, the hardest part is in the best position—that exposed to wear. If the cavity be double the depth I have described, then take half as much of the spongy metal as would serve for its completion, and thoroughly compress it, but with as little burnishing of the surface as possible. The cavity will then be in the position of the one I first described, and may be treated in the subsequent stage of the operation similarly. By adopting this plan a more solid plug is gained, than if a sufficient quantity of gold for completing the operation had been introduced at once. This method would not be very effective with gold foil, but with the sponge the welding is so perfect, that there is no chance of the upper part coming out or crumbling to pieces; indeed, it is exceedingly difficult to get a plug made with sponge gold out of its cavity, unless by drilling. In this respect it resembles a plug made with amalgam.

If the cavity be broad and shallow, the further side may be first filled by pressing the gold in that direction, before too much pressure has been applied on the surface of the plug. When the further half has been filled, and vertical wall of gold left, the other half may be filled. The two halves of the plug will weld, if in making the wall of the first half, the surface has not been burnished.

An equally good plug may be made, by introducing a mass of gold sponge, and after compressing it moderately, making a hole in the center, and from this, compressing the stopping towards the walls of the cavity. By this method the cavity becomes lined with a cup of solid metal, the center of which can be readily filled, and the plug made sound.

In large angular cavities, such as are sometimes found in the molar teeth, more especially in those of the lower jaw, a good plug may be made by forcing the gold into the angles; thus reducing the cavity to a cylindrical one. The bottom may then be filled to within the tenth of an inch of the surface, and the remaining part completed. With gold foil, the filling the angles of a cavity is often troublesome, but with spongy gold it is an easy matter.

When the compressing instrument ceases to take effect, in fact, when no further consolidation of the metal can be effected —the plug should project above the edges of the cavity, so, that when the surface has been cut or filed away, and further compression is attempted, the plug may not sink below the margins of the cavity.

A sharp wedge-shaped instrument should at this period of the operation be used, and if any parts are found which can be pierced, these should be filled with a little of the leaf gold.

In finishing, all the tool-marks should be removed, the margin of the plug made perfectly level with the surrounding part of the tooth, and with the centre a little elevated. It should, lastly, be brightly burnished.

In using the new forms of gold, it is desirable to bear in mind, that the surfaces of the metal should be handled as little as possible, otherwise, they will become coated with extraneous matter, which will interfere with the welding. Neither should the stock of gold be exposed to the atmosphere. It may be conveniently kept in a wide mouth bottle, which admits of being well corked. Should, however, the metal lose its softness or its welding properties, it may be restored to its former condition by annealing over a spirit lamp.

In selecting the gold for making a plug, I have usually employed the sponge for filling the cavity, and the leaf for stopping the cavity made by the wedging instrument in the partially completed plug.

The two kinds of gold manufactured by Mr. Makins, may, I believe, be obtained of Mr. Ash of Broad-street, Golden-square; and I would urge upon dentists, the expediency of giving them a fair trial. At present but little has been made; it is, therefore, probable, that with further experience, many improvements in the condition of the gold may be made. But should it be shown that better spongy and leaf gold cannot be produced than that with which Mr. Makins has supplied me, even then, I think, there is great cause for congratulation, for I believe it will be found that better plugs can be made with these forms, than can, on the average, be made with the gold foil prepared by hammering in the usual manner.—*Med. Gaz.*

